# Bio-Fabricated Silver Nanoparticles from the Leaf Extract of the Poisonous Plant, *Holigarna arnottiana*: Assessment of Antimicrobial, Antimitotic, Anticancer, and Radical-Scavenging Properties

**DOI:** 10.3390/pharmaceutics15102468

**Published:** 2023-10-15

**Authors:** Anthyalam Parambil Ajaykumar, Ovungal Sabira, Valiyaparambil Sivadasan Binitha, Sudhir Rama Varma, Anjaly Mathew, Kodangattil Narayanan Jayaraj, Pandikkadan Ayyappan Janish, Koladath Vasu Zeena, Padannappurath Sheena, Veena Venugopal, Priyanka Palakkapparambil, Aswathi

**Affiliations:** 1 Division of Biomaterial Sciences, Department of Zoology, Sree Neelakanta Government Sanskrit College, Pattambi, Palakkad 679303, Kerala, India; sabiraovungal@gmail.com (O.S.); janishpa@sngscollege.org (P.A.J.); zeenasalim@gmail.com (K.V.Z.); sheenapnarayan@gmail.com (P.S.); veenarun86@gmail.com (V.V.); priyapkd1995@gmail.com (P.P.); achuthy7@gmail.com (A.); 2 Department of Zoology, Sree Narayana College, Nattika, Thrissur 680566, Kerala, India; binisncn@gmail.com; 3 Clinical Sciences Department, Centre for Medical and Bio-Allied Health Sciences Research, Ajman University, Ajman P.O. Box 346, United Arab Emirates; s.varma@ajman.ac.ae; 4 Department of Chemistry, Sree Neelakanta Government Sanskrit College, Pattambi, Palakkad 679303, Kerala, India; anjalymathew4@gmail.com; 5 Basic Sciences Department, Centre for Medical and Bio-Allied Health Sciences Research, Ajman University, Ajman P.O. Box 346, United Arab Emirates

**Keywords:** *Holigarna arnottiana*, silver nanoparticles, radical scavenging, anticancer activity, LC-MS analysis

## Abstract

This study presents a novel approach to fabricate silver nanoparticles (AgNPs) using the poisonous plant, *Holigarna arnottiana* leaf extract. The formation of AgNPs was confirmed by a color change from green to dark brown and validated by UV analysis. FTIR analysis identified functional groups on the AgNPs, while Zeta potential analysis assessed their stability. TEM analysis established an average diameter of 18 nm and a spherical morphology for the nanoparticles. LC MS analysis coupled with database searches revealed the presence of diverse bioactive compounds, including flavonoids, nucleotides, dipeptides, enzymes, and glycosides. These compounds are postulated to act as reducing agents in the leaf extract-mediated synthesis process. Moreover, the bio-fabricated AgNPs exhibited noteworthy anticancer properties against DLA cells. In addition, AgNPs displayed substantial antimitotic effects in an assay involving *Allium cepa* root cells. These findings underscore the potential of the AgNPs as cytotoxic agents. The biosynthesized AgNPs showed antimicrobial activity against various bacterial pathogens, including *Escherichia coli*, *Klebsiella pneumoniae*, and *Staphylococcus aureus*. Furthermore, the AgNPs exhibited outstanding radical-scavenging properties in the DPPH assay, suggesting their potential application in antioxidant therapies. The study collectively highlights the successful synthesis of AgNPs through a green, biocompatible approach, and demonstrates their promising potential for anticancer, antimitotic, and radical-scavenging applications.

## 1. Introduction

The nanotechnology field has recently experienced significant advancements in the synthesis of metal nanoparticles because of the broad range of applications for nanoparticles, from medicine to environmental remediation. Among these nanoparticles, silver nanoparticles (AgNPs) have attracted the most interest due to their unique physicochemical properties that make them suitable for biological, catalytic, and antibacterial applications. However, the environment is often compromised by the use of hazardous chemicals and energy-intensive processes in current chemical and other AgNP synthesis methods. To solve these issues and advance environmentally friendly nanoparticle synthesis, researchers have increasingly concentrated on green synthesis methods that utilize natural resources like plant extracts to produce AgNPs [1,2,3,4].

The endemic tree *Holigarna arnottiana* (Family: Anacardiaceae) is a large tree, that can reach a height of up to 35 m. Its bark exhibits fine fissures and pustules, while its branches are robust, circular, and devoid of hair. Its leaves are simple, alternate, arranged spirally and clustered at twig ends. The tree is endemic to The Western Ghats [5]. It is used as a medicinal plant and as a folk medicine by village people. The plant is used to treat a wide range of discomforts like fever, cough, skin diseases, rheumatism, snakebite, jaundice, dysentery, etc. [6]. *Holigarna* has a scanty exudate of colorless sap which becomes black on drying. The sap of the tree causes various severe anaphylactic skin responses [7]. Mane and co-workers made an investigation, aiming to obtain chromosomal information in *Holigarna* without previous cytological information and find out the karyotype formula as 21 m + 8 sm [8]. sm [8]. GC-MS analysis of leaf and bark extracts uncovered the presence of around 20 distinct compounds known for their beneficial biological properties. Additionally, phytochemical investigations confirmed the existence of compounds like coumarins, saponins, tannins, reducing sugars, and others. Notably, the extract was also found to contain urushiol, a substance with the potential to induce allergic reactions [9]. *Holigarna* shows strong antibacterial activity towards several Gram positive and Gram negative pathogenic bacteria, and against human and shrimp pathogenic bacteria, and a GC-MS analysis was used to elucidate its antimicrobial principles. Based on the findings, it could be inferred that *H. arnottiana* could be used as a reliable source for developing bio-therapeutics for shrimp and humans in the future [10]. Contradictory to their strong antimicrobial activity, fungal isolates were resistant to the plant-derived extracts of *H. arnottiana* and showed specific activity against bacteria only. The organic extracts had more antibacterial activity as compared to the water extracts [11]. Recent studies unveiled their antioxidant activity using the superoxide radical scavenging method and the Hydroxy radical assay. Strong cytotoxic activity was detected using the MTT assay and DLA assay [12,13].

The plant kingdom provides a rich source of reducing and capping agents for the environmentally friendly synthesis of nanoparticles due to its vast variety of secondary metabolites. The flavonoids, terpenoids, and phenolic chemicals found in this plant are recognized for their wide range of bioactive substances, which have a variety of medicinal qualities. In the current study, the potential bioactivity of silver nanoparticles produced from *H. arnottiana* leaf extract is explored. The synthesis process makes use of the inherent reduction power of the leaf extract of *H. arnottiana* and is thus an appealing replacement for conventional methods because it is eco-friendly, free of dangerous chemicals, and energy-efficient. The natural capping agents included in the plant extract are expected to improve the stability and biocompatibility of the resulting silver nanoparticles, which may boost their potential use in biomedicine.

The primary objective of this research is to assess the synthesized silver nanoparticles by characterizing them and examining their potential in various aspects, including anticancer, antimicrobial, cytological aberration, and scavenging properties. In the realm of oncology, there is an increasing necessity for novel anticancer agents that offer enhanced effectiveness while minimizing side effects. Moreover, in light of the escalating challenge of antibiotic resistance, there is a growing demand for alternative antimicrobial agents, and silver nanoparticles have displayed promise in addressing this concern. Additionally, the antioxidant and free-radical-scavenging properties of nanoparticles hold the potential to combat disorders associated with oxidative stress, thus creating new avenues for their utilization in antioxidant therapy.

## 2. Materials and Methods

### 2.1. Preparation of Holigarna arnottiana Leaf Extract

**Figure 1:**
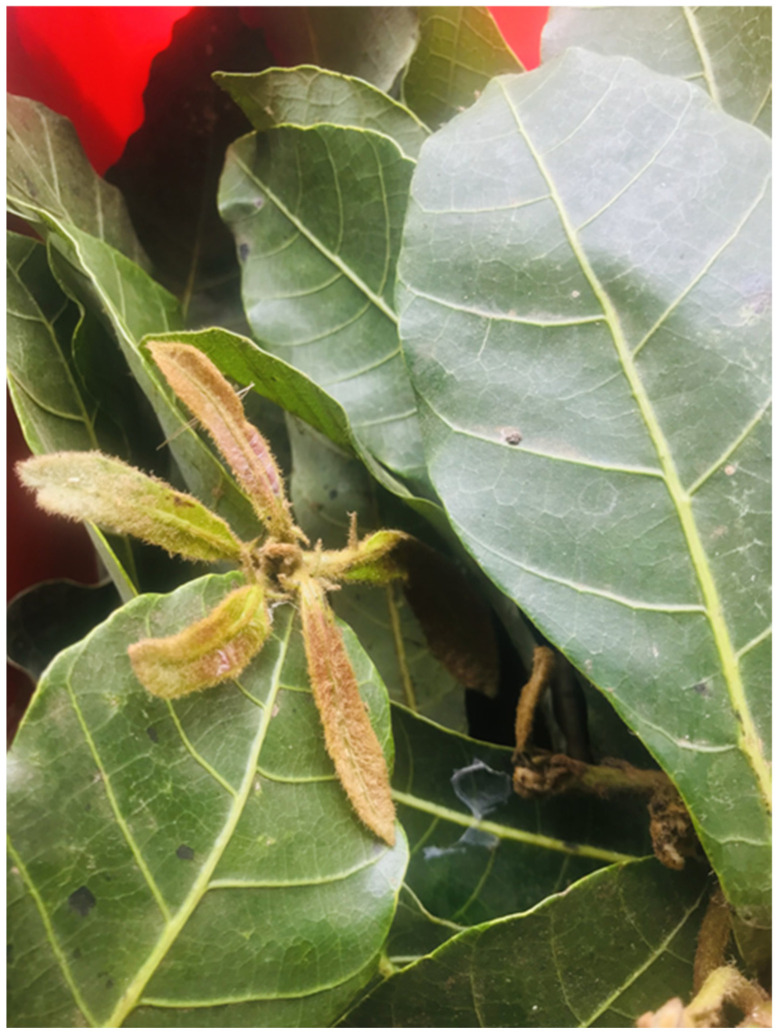
*H. arnottiana*.

Fresh leaves of *H. arnottiana* (Figure 1) were locally gathered from the Malappuram district (located at 75°58′25.7″ E) in the Indian state of Kerala. An expert from the Department of Botany at the University of Calicut, India, validated their identification to ensure accuracy. The leaves were subjected to thorough washing with deionized water through repeated cycles. Subsequently, 10 g of the sliced leaves was placed into a 250 mL beaker, and 200 mL of distilled water was added. Subsequently, the beaker underwent microwave irradiation for approximately 2 min, utilizing microwave radiation at a frequency of 2.45 GHz and a microwave power of 350 W. The pale-green filtrate was acquired by centrifuging the mixture at 6000× *g* for 2 min. Afterward, it was placed in a refrigerator at a controlled temperature of 5 °C to ensure its preservation for further investigation.

#### LC MS Analysis of Leaf Extract of *H. arnottiana*

Metabolite identification from the methanol extract of *H. arnottiana* leaf was assessed using an LC-ESI-Q-TOF-MS system (Agilent Technologies 6550 i-Funnel, Santa Clara, CA, USA) with a G4220B pump, G4226A autosampler, and G1316C, along with a polydiode array detector (PAD). The elution solution was composed of a gradient mixture of 0.1% formic acid in water (A) and acetonitrile (B), operating at a flow rate of 0.30 mL/min. The gradient began at 95% A and 5% B, transitioning to 5% A and 95% B over 25 min, before returning to the initial composition (95% A: 5% B) within 10 min, maintained for 5 min. The mass spectrometry (MS) analysis was conducted using ESI in the positive ionization mode. The MS source settings were as follows: capillary voltage of 3500 V, gas temperature set at 250 °C, drying gas flow rate of 13 L/min, sheath gas temperature of 300 °C, sheath gas flow at 11 units, nebulizing gas pressure at 35 psig, fragment set at 175 V, skimmer at 65 V, octopole RF peak at 750 V, and a mass range for detection spanning m/z 50 to 1000. The Mass Hunter software, Version: Q-TOF B.05.01 (B5125.3), developed by Agilent Technologies, was employed to analyze the mass spectra and chromatograms.

### 2.2. Synthesis of Silver Nanoparticles Using H. arnottiana Leaf Extracts

Silver nitrate (AgNO_3_) was purchased from Merck Chemicals (99.8%) and used for the biosynthesis of silver nanoparticles. Silver nanoparticles (AgNPs) were synthesized through a method involving the heating of a solution comprising AgNO_3_ and *H. arnottiana* leaf extract in a common household microwave oven (LG-MS-2029 UW). The reaction mixture was prepared by mixing 10 mL of the leaf extract combined with 50 mL of a 0.001 M silver nitrate solution. Subsequently, the reaction mixture was put into a domestic microwave oven operating at 2.45 GHz and 350 W for approximately 10 min. An alteration in color, progressing from pale green to pale yellow and eventually to deep brown, was observed, indicating the successful formation of AgNPs. The AgNPs synthesized and mediated by *H. arnottiana* were isolated by being subjected to centrifugation at 12,000 rpm for 10 min. This procedure was repeated two times to eliminate any residual-free silver. Subsequently, the resultant green-synthesized AgNPs were dried and stored at a temperature of 4 °C until they were needed for future analysis.

### 2.3. Characterization of AgNPs

The initial confirmation of AgNP formation was assessed through UV–vis spectroscopy. The identification of functional groups linked to the *H. arnottiana*-mediated AgNPs was accomplished via FTIR (PerkinElmer, Waltham, MA, USA) analysis. The stability assessment of the AgNPs was conducted using zeta potential measurements. The dimensions and morphology of the AgNPs were determined through TEM (JEOLJEM-2100 microscope, Montgomery, AL, USA) imaging. Collectively, these analytical techniques contributed to an in-depth characterization of the synthesized AgNPs, offering insights into their optical, structural, surface chemical, and colloidal attributes.

### 2.4. Cyclic Voltammetry Analysis

The electrochemical behavior of *H. arnottiana* leaf extract was investigated through cyclic voltammetry (CV). The CV measurement was conducted using a digital Ivy Potentiostat (Model No. DY2000EN) equipped with DY 2000 software. A three-electrode electrochemical arrangement was employed, consisting of a glassy carbon electrode (GCE) as the working electrode, a platinum wire serving as the counter electrode, and an Ag/AgCl reference electrode for monitoring CV signals. The CV measurements were carried out in *H. arnottiana* leaf extract containing a solution of 0.1 M phosphate buffer (pH = 7.4) and 0.1 M KCl as a supporting electrolyte.

### 2.5. Antimicrobial Effects of AgNPs on Bacterial Pathogens

The antimicrobial properties of green synthesized AgNPs were tested against four different bacterial pathogens, such as *Escherichia coli*, *Klebsiella pneumoniae*, *Staphylococcus aureus*, and using the agar well diffusion method [14,15,16]. The four bacteria strains were applied on Mueller–Hinton agar using a sterilized cotton swab (MHA). A sterile blank antimicrobial susceptibility disc was used in the test. The discs were loaded with 10 µL of AgNPs as an experiment (represented as A); leaf extracts were utilized as a control (represented as C), and the antibiotic was tetracycline employed as a positive control (represented as B). The discs were then placed on the agar plate and incubated at 37 °C for 24 h. The inhibitory zone was examined after 24 h of incubation.

### 2.6. Antimitotic Assay

Various chromosomal abnormalities were studied in healthy young *Allium* bulbs. They were grown in synthesized AgNPs used for this purpose. The control was grown in distilled water. Using the squashing method, the squash preparation was performed, and using the camera-attached microscope, the chromosome images were taken at 40× magnification.

### 2.7. Cytotoxicity of AgNPs in Cancer Cell Lines

The evaluation of the cytotoxicity of biofabricated AgNPs involved an investigation into its impact on Dalton’s lymphoma ascites cells (DLA cells). The analysis was carried out in Amala Cancer Research Institute, Thrissur, Kerala, India. The primary objective of this study was to establish cell viability, which was accomplished through the utilization of the trypan blue exclusion technique. Tubes containing varying concentrations of biosynthesized AgNPs (12.5 µg/mL, 25 µg/mL, 50 µg/mL, 100 µg/mL, and 150 µg/mL and 200 µg/mL) were meticulously prepared. Each tube was combined with a viable cell solution comprising 1 × 10^6^ cells in 0.1 mL. In order to achieve a total volume of 1 mL, phosphate buffer solution was added to each tube, except for the control tube which solely contained the cell suspension. Following this, the resulting mixture was subjected to an incubation period exceeding three hours at a temperature of 37 °C.

Afterwards, the cell solution was merged with 0.1 mL of a 1% trypan blue solution. The mixture was allowed to settle for 2–3 min, after which it was transferred to a hemocytometer. Notably, the trypan blue imparted a blue hue to the deceased cells, while the viable cells remained devoid of staining. Subsequent to this, the quantities of stained and unstained cells were directly counted. This process facilitated the determination of both the total number of stained cells and the number of unstained, viable cells [17]. The percentage of cytotoxicity was calculated by using the following formula. Percentage of cytotoxicity = number of dead cells/number of live cells + number of dead cells × 100.

### 2.8. Antioxidant Activity

The DPPH free radical scavenging assay was used to assess the antioxidant properties of various substances. A brand-new DPPH solution was made in methanol, and the precise beginning concentration was determined spectrophotometrically from a calibration curve. At 517 nm, the absorbance reduction was seen. The following equation was used to compute the DPPH radical-scavenging capability. S% = [(A control − A sample)/A control] × 100.

The linear regression equation was used to determine the sample concentration needed to induce 50% scavenging activity (IC 50 value) from the graph.

## 3. Result and Discussion

### 3.1. Characterization of Metabolites in the Leaf Extract by LCMS Analysis

**Figure 2:**
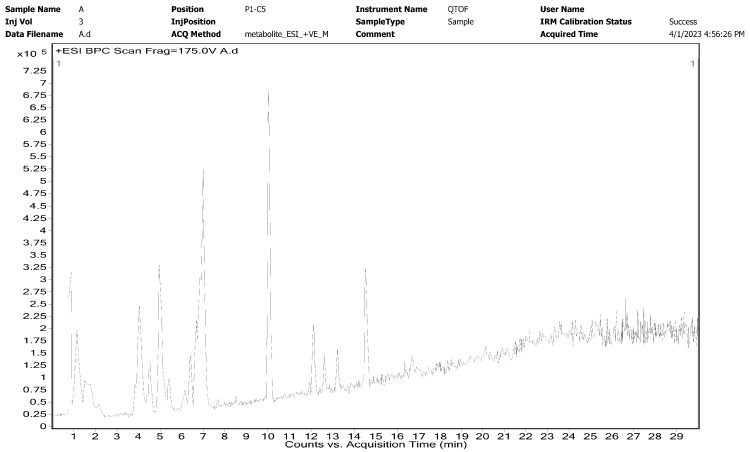
LC MS profile of leaf extract of *H. arnottiana*.

The LC MS analyses of the secondary metabolites present in the leaf extract of *H. arnottiana* are presented in the Figure 2. The results unveiled a diverse range of compounds in the analysis. These included fatty acids such as Propanoic acid and 2-Hydroxypelargonic acid, flavonoids like Quercetin, Quercetin 3-beta-L-arabinopyranoside, Lonchocarpene and Robinetin 7-glucoside, phenolic compounds such as Deshydroxypyrogallin-4-carboxylic acid, as well as peptides such as hydroxyphenylalanyl-aspartic acid. Furthermore, the leaf extract consist of an enzyme like 2-Hydroxymethylserine, specific amino acid derivatives such as 2-Hydroxymethylserine, nucleotide sugar like UDP-2-acetamido-4-amino-2,4,6-trideoxyglucose, and compounds like naphthoquinones (e.g., juglalin) and terpenes. Each of these identified molecules within the leaf extract of *H. arnottiana* possesses distinct chemical properties that could potentially play a role in reducing and stabilizing silver ions during the synthesis of nanoparticles.

### 3.2. UV Analysis

**Figure 3:**
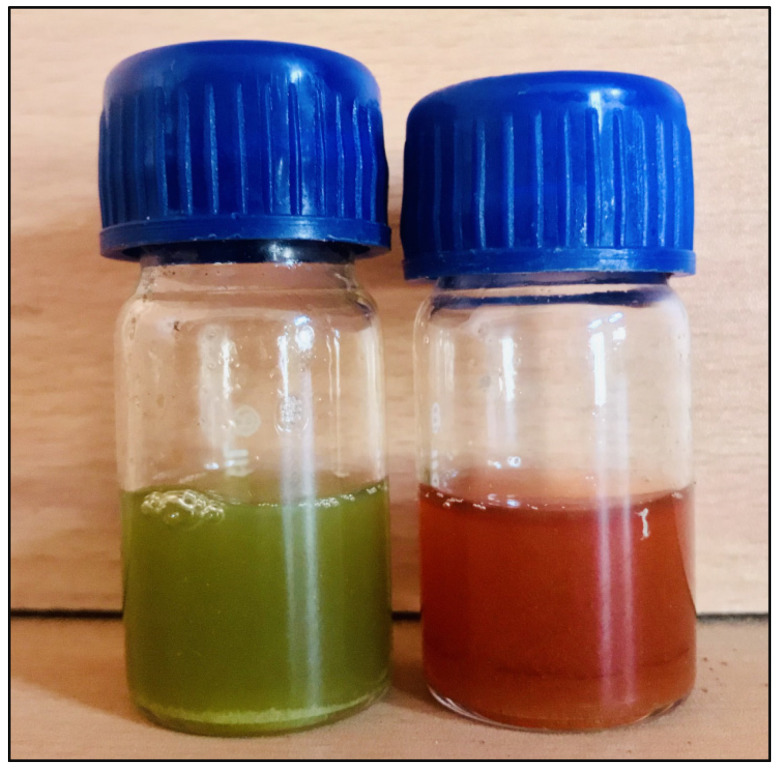
The successful synthesis of AgNPs was evidenced by a visual transition from a pale green to a pale yellow shade, followed by a deep brown coloration.

**Figure 4:**
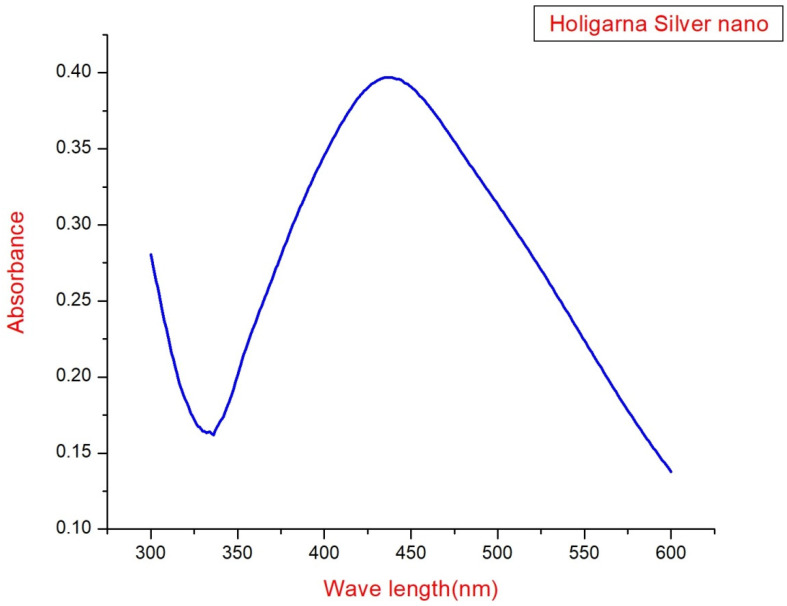
UV-Vis spectrum of AgNPs.

During the biological synthesis of AgNPs, a noticeable shift in solution color from pale green to brown serves as a prominent indicator of AgNP formation (Figure 3. The findings of the UV-Vis analysis are presented in Figure 4. The result showed a prominent peak at 448 nm in the spectrum, which signifies the Surface Plasmon Resonance feature resulting from the oscillation of surface electrons of silver metal within an electromagnetic environment, which indicates that the effective formation of AgNPs through the utilization of leaf extract is validated. Earlier studies with green synthetic silver nano from *Ocimum sanctum* and marigold extract found that the maximum absorbance ranged from 400 to 500 [18,19].

### 3.3. FTIR Analysis

**Figure 5:**
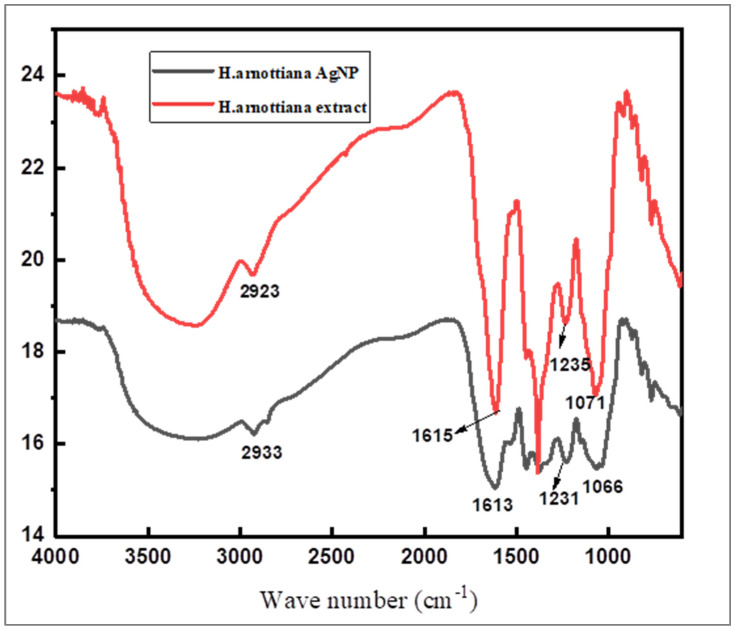
FTIR spectra of leaf extract and AgNPs.

Using FTIR spectroscopy, the dual function of leaf extract as a reducing and capping agent was established. The FTIR spectra of the powders of the aqueous extract of *H. arnottiana* and AgNPs were recorded using the KBr pellet method with a Perkin Elmer spectrophotometer in the range of 400–4000 cm^−1^. Figure 5 shows the FTIR spectra of *H. arnottiana* leaf extract and AgNPs synthesized using *H. arnottiana*. The two spectra may be seen to have small differences in intensity, making them practically identical.

A peak at 2933 cm^−1^ of the leaf extract arises from the C–H stretching vibrations. An amide I band appears at 1615 cm^−1^. The peak at 1383 cm^−1^ is assigned to the –COO stretching from amino acid groups. The band at 1071 cm^−1^ and 822 cm^−1^ is assigned to C–C stretching vibration and the aromatic –C–H bending vibrations, respectively. This implies that terpenoids, flavonoids, phenols, and phytosterols found in the *H. arnottiana* leaf extract play a role in the synthesis and stabilization of nanoparticles [20]. These molecules have been identified in the *H. arnottiana* leaf extract through LC MS analysis (as shown in Figure 2 and Table 1). It can be inferred that biomolecules from the leaf extract containing active functional groups are capable of reducing silver ions into metallic AgNPs. Furthermore, there is a proposition that these functional groups may combine with silver, creating a protective capping layer around the AgNPs, preventing their aggregation. Previous research has demonstrated that carbonyl, ester, amino, hydroxyl, and phenolic groups effectively bind with the noble metals through coordination and polar interactions [21]. The small variation in the intensity and position of the peaks in the spectrum of nanoparticles is due to the coordination of phytochemicals with metals [22].

### 3.4. Zeta Potential Analysis

**Figure 6:**
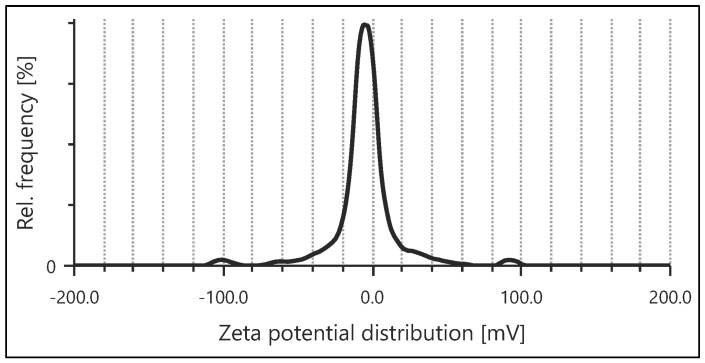
Zeta potential distribution of AgNPs.

Zeta potential analysis serves as a pivotal factor that characterizes the surface electrostatic charge of nanoparticles. In this study, the AgNPs that were synthesized displayed a zeta potential value of −8.7 mV (Figure 6). The negative zeta potential value (−8.7 mV) implies a state of repulsion among the AgNPs, potentially bolstering their stability in suspension. This outcome contributes to the effective dispersion and compatibility of AgNPs within physiological surroundings. The findings enrich our comprehension of how AgNPs synthesized with the assistance of *H. arnottiana* behave in biological and environmental settings, and how they can be further applied in the biomedical field. According to a current study, AgNPs are more stable. The capping of the extract’s polyphenolic contents may be the cause of the high negative potential value [23,24].

### 3.5. Cyclic Voltammetry Analysis

**Figure 7:**
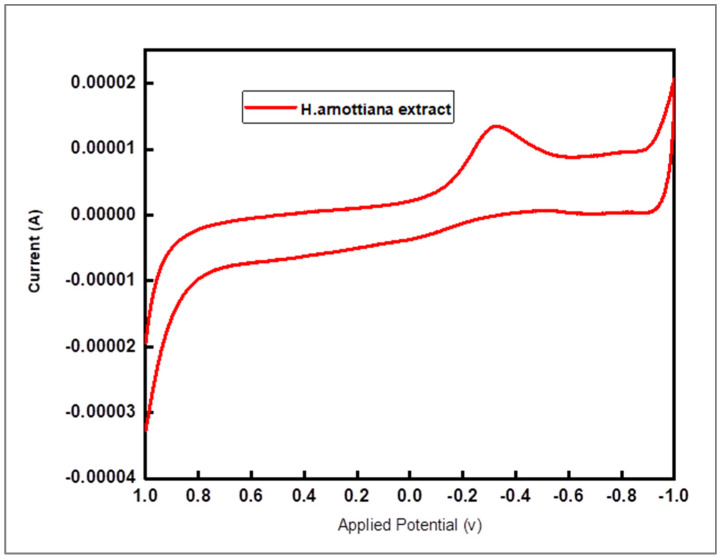
Cyclic voltammetry of leaf extract of *H. arnottiana*.

Cyclic voltammetry (CV) was utilized to explore the electrochemical behavior of the leaf extract derived from *H. arnottiana*. A single anodic peak current appeared in the cyclic voltagram of the plant extract at a potential of −0.318 V (Figure 7). CV analysis confirmed the effective reduction capacity of *H. arnottiana* leaf extract, facilitating the conversion of Ag ions into AgNPs. Silver nanoparticles synthesized via a green synthesis approach displayed no reducing properties, as shown in Supplementary Figure S1. This finding inspires additional investigation to fully realize the potential of natural resources for a range of applications and shows great promise for environmentally friendly and sustainable nanoparticle synthesis processes. Comparable outcomes were achieved via the CV analysis using *Uverianarum* and *Mimosa albida* leaf extract, as reported in a previous study [25,26].

### 3.6. Transmission Electron Microscopy Analysis

**Figure 8:**
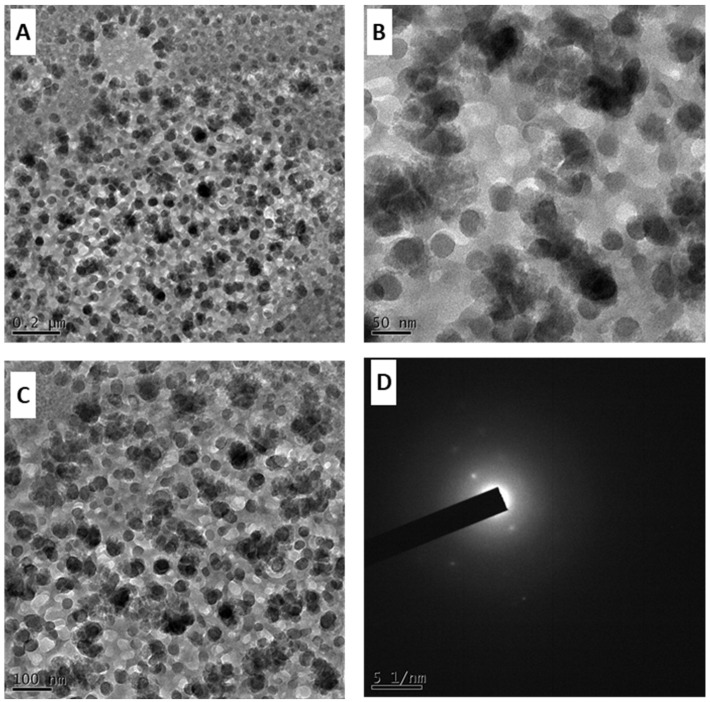
Transmission Electron Microscopy (TEM) images of AgNPs. Scale bars in the figures represent 0.2 µm (**A**), 20 nm (**B**), 100 nm (**C**), and 5 1/nm (**D**), respectively.

The leaf extract of *H. arnottiana* was harnessed for the synthesis of AgNPs, which were subsequently examined using Transmission Electron Microscopy to gain insights into their shape and size. These variables hold substantial influence over their attributes and potential applications. The investigation disclosed that the AgNPs created through the utilization of *H. arnottiana* leaf extract displayed a spherical shape, with an average diameter measuring 18 nm (Figure 8), consistent with earlier research findings [27]. This choice of size holds critical importance, as nanoparticle dimensions yield a substantial impact on a multitude of traits, encompassing optical characteristics, magnetic responses, and catalytic capabilities. Furthermore, due to their nano-scale dimensions, AgNPs possess the potential to permeate cellular structures, rendering them robust candidates for applications spanning targeted therapeutic interventions, imaging modalities, and drug delivery methods. Ecologically sound plant extracts contain biomolecules that serve as capping and reducing agents to create stable, shape-controlled nanoparticles. Biomolecules including phenolics, terpenoids, and polysaccharides are among the main substances that have been documented to alter the reduction and capping of nanoparticles [28,29].

### 3.7. Antibacterial Assay

**Figure 9:**
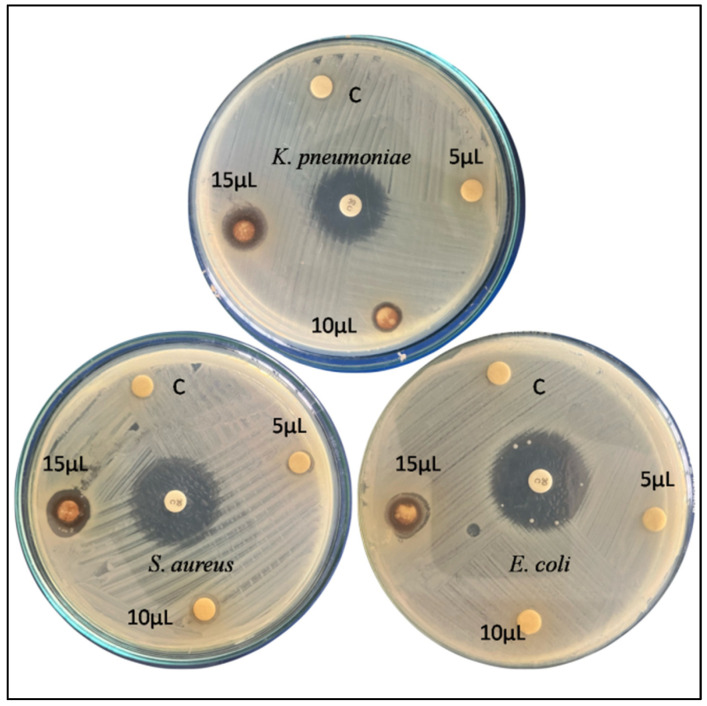
Antibacterial activity of AgNPs against *K. pneumoniae*, *S. aureus*, and *E. coli*. Three distinct concentrations (5 µL, 10 µL, and 15 µL) of AgNPs are evaluated, along with a control (C) that represents the leaf extract of *H. arnottiana*. The antibiotic, tetracycline, was applied at the central portion of the disc as a positive control.

The disc diffusion assay was used in the current investigation to look into the antibacterial capabilities. It was evaluated how AgNPs affected the development of both Gram-positive (*Staphylococcus aureus* and *Escherichia coli*) and Gram-negative (*Klebsiella pneumoniae*) bacteria. Control plates loaded with plant extract (5 μL) exhibited a zone of inhibition (ZOI) measuring 7 mm. However, when filter papers infused with AgNP concentrations of 5 μL, 10 μL, and 15 μL were used, the diameter of the inhibition zone showed ranges of 10 mm, 15 mm, and 18 mm, respectively, for *K. pneumoniae* (as depicted in Figure 9). In the case of *S. aureus*, the ZOI for the control (5 μL) was 8 mm, while filter papers containing 5 μL, 10 μL, and 15 μL of AgNPs exhibited inhibition zone diameters of 10 mm, 14 mm, and 16 mm, respectively. As in the case of *E. coli*, the ZOI for the control shows a mortality of 5 mm and 7 mm, 9 mm, and 12 mm for 5 μL, 10 μL, and 15 μL of AgNPs. As the concentration of AgNPs rises, the antibacterial activity of AgNPs shows a dose-dependent response, with a stronger activity against *K. pneumoniae* as compared to *S. aureus* and *E. coli.* It shows that silver nanoparticles have a potent antibacterial impact, as numerous scientists have already found [17,25,30,31,32,33,34,35,36,37,38,39].

**Figure 10:**
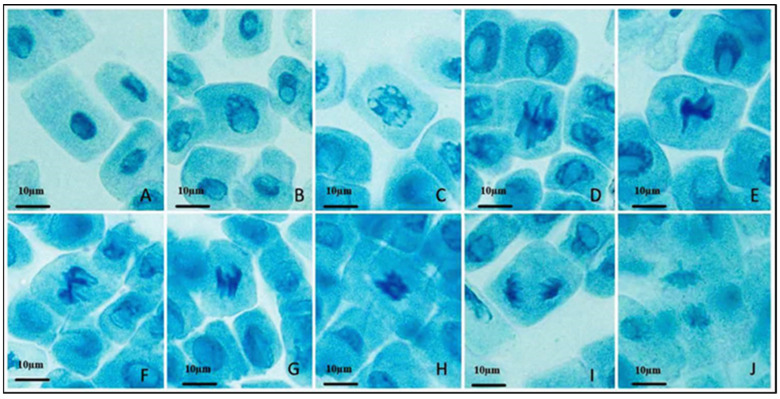
Chromosomal effect of green synthesized AgNPs. (**A**) Normal nuclei. (**B**,**C**) Disintegrated nuclei. (**D**) Normal metaphase. (**E**–**H**) Laggard metaphase. (**I**) Normal Anaphase. (**J**) Laggard anaphase.

The effects of green synthesized AgNPs in the *Allium cepa* root tip cells provide valuable insights into the potential cytological impacts of AgNP exposure [40,41,42,43]. The microscopic examination of the root tip cells revealed distinct effects on chromosomal behavior upon exposure to AgNPs (Figure 10). In the figure, the control group displayed regular nuclei, indicating the fundamental condition of chromosome (A) division when AgNPs were absent. Images (B and C) exhibit nuclei disintegration: the presence of AgNPs seemed to cause nuclei disintegration in certain cells (B and C). This occurrence implies that AgNPs might potentially disrupt the structural integrity of nuclei, resulting in irregular cellular states. (D) Typical Metaphase: in the control group, cells depicted a normal metaphase, signifying the correct alignment of chromosomes on the metaphase plate—a pivotal phase in chromosome separation. (E, F, and G) Delayed Metaphase: intriguingly, cells subjected to AgNP exposure presented lagging chromosomes during metaphase (E, F, G, and H). This observation implies a possible delay or hindrance in chromosome alignment, which could lead to the uneven distribution of genetic material during cell division. (I) Standard Anaphase: control cells exhibited regular anaphase, characterized by the accurate separation of sister chromatids towards opposite poles of the cell (J). This demonstrates a degree of similarity to other works [44,45,46]. A similar cytological analysis employing *H. arnottiana* leaf extract did not reveal significant alterations in the cell division of *Allium cepa* root tips (Supplementary Figure S2).

### 3.8. Cytotoxicity Effect of AgNPs

**Figure 11:**
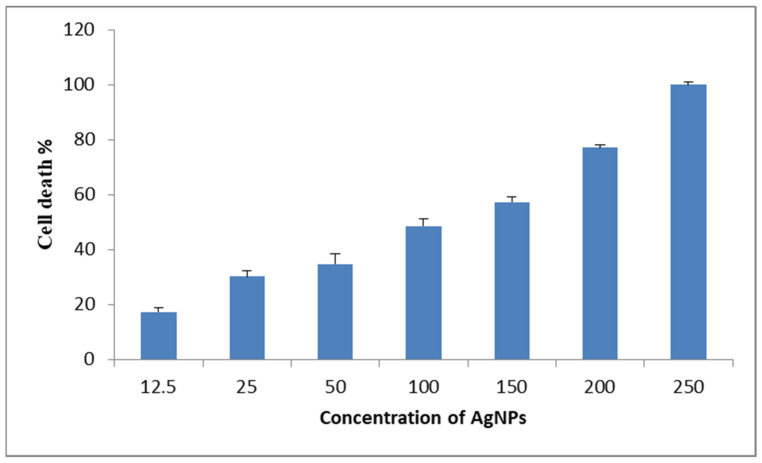
Anticancer property of AgNPs on DLA cancer cells.

The anticancer activity of green synthesized AgNPs was estimated on DLA cells via a trypan blue assay (Figure 11). At a silver concentration of 12.5 µg, a 17.4% reduction in cell viability was observed, indicating a mild impact. As the AgNP concentration increased to 25 µL, 50 µL, and 100 µL, the cytotoxic effects escalated to 30.3%, 34.8%, and 48.7%, respectively. Notably, at AgNP concentrations of 150 µL and 200 µL, the cytotoxicity reached a significant level with a substantial reduction in cell viability at 57.4% and 100%. This result underscores the potential of AgNPs to induce strong cytotoxic responses at elevated concentrations, aligning with previous studies that have reported the dose-dependent cytotoxicity of nanoparticles [47]. The results of this study underscore the concentration-dependent cytotoxic effects of AgNPs on cell viability. The significant reduction in cell viability at higher AgNPs concentrations aligns with the existing understanding of nanoparticle toxicity trends [48,49,50,51,52,53,54]. These findings emphasize the importance of careful consideration and dose regulation when utilizing AgNPs in biomedical applications and highlight their potential for inducing cytotoxic responses at elevated concentrations. In cells exposed to AgNPs, a laggard anaphase was observed. This phenomenon implies that AgNPs might have interfered with the proper separation of chromatids during anaphase, potentially leading to unequal genetic distribution.

### 3.9. Antioxidant Activity

Free radical scavenging activity was measured using the DPPH method, and various concentrations (20 µL, 40 µL, 60 µL, 80 µL, and 100 µL) of AgNPs were utilized for the analysis. The solution was exposed to UV spectroscopy for examination after an incubation time of 30 min in complete darkness. At 517 nm, the greatest amount of electrons was absorbed by DPPH-free radicals. The results show evidence of concentration-dependent scavenging activity. The sample concentration needed to provide 50% scavenging activity (EC50 value) was found to be 80 µL. The assessment of scavenging activity using *H. arnottiana* leaf extract displayed a notably higher EC50 value when compared to AgNPs synthesized through green methods, as depicted in Supplementary Figure S3. The current study confirms earlier findings by demonstrating a dose-dependent increase in the scavenging activity of AgNPs [25,39,55,56,57,58,59].

## 4. Conclusions

In conclusion, the green synthesis of silver nanoparticles using *Holigarna arnottiana* leaf extract represents a promising approach that aligns with the principles of sustainability and eco-friendliness. The multifaceted bioactivities of these nanoparticles, including anticancer, antimicrobial, and scavenging properties, hold immense potential for future biomedical and environmental applications. This research aims to contribute valuable insights into the development of novel and sustainable nanomaterials with significant biomedical relevance.

## Figures and Tables

**Table 1:** Different compounds identified from the leaf extracts of *H. arnottiana* via LC MS analysis.

SL. No	Name of Molecule	Retention Time (RT)	Mass	Chemical Formula	Class of Compound
1	7-Deshydroxypyrogallin-4-carboxylicacid	0.941	248.0343	C_12_H_8_O_6_	Phenolic compound
2	2-Hydroxymethylserine	1.092	135.0525	C_4_H_9_NO_4_	Derivative of the amino acid serine
3	ProLeu (prolylleucine)	1.351	228.1447	C_11_H_20_N_2_O_3_	Dipeptide
4	2-Hydroxypelargonic acid	2.10	174.1256	C_9_H_18_O_3_	Fatty acid
5	Lonchocarpenin	5.024	448.1876	C_27_H_28_O_6_	Flavonoid
6	UDP-2-acetamido-4-amino-2,4,6-trideoxyglucose	5.396	590.1023	C_17_H_28_N_4_O_15_P_2_	Nucleotide sugar
7	Robinetin7-glucoside	6.408	464.0897	C_21_H_20_O_12_	Flavonoid
8	Juglalin	6.488	418.0895	C_20_H_18_O_10_	Naphthoquinone,
9	Propanoic acid	6.495	248.0676	CH_3_CH_2_COOH	Short-chain fatty acid
10	Quercetin 3-beta-L-arabinopyranoside	6.706	434.079	C_20_H_18_O_11_	Flavonoid glycoside
11	Quercetin	6.779	302.0387	C_15_H_10_O_7_	Flavonoid
12	Asn-Trp-OH	7.014	426.1121	C_20_H_18_N_4_O_7_	Dipeptide
13	Dihydrophaseicacid4-O-beta-D-glucoside	13.278	444.1992	C_21_H_32_O_10_	Glycoside

## Data Availability

The data generated and analyzed during the current study are available from the corresponding author upon reasonable request.

## Supplementary Materials

The following supporting information can be downloaded at: https://www.mdpi.com/article/10.3390/pharmaceutics15102468/s1, Figure S1: CV data of AgNPS synthesized using *H. arnottiana*; Figure S2: The Effect of Leaf Extract and Silver Nanoparticles on Mitotic Activity in Onion Root Tip Cells in the Context of Chromosomal Aberrations; Figure S3: DPPH activity of leaf extract of *H. arnottiana*.
