# Supplementary material for: Bio-Fabricated Silver Nanoparticles from the Leaf Extract of the Poisonous Plant, Holigarna arnottiana: Assessment of Antimicrobial, Antimitotic, Anticancer, and Radical-Scavenging Properties
Source: Pharmaceutics. 2023 Oct 15;15(10):2468. doi: 10.3390/pharmaceutics15102468 (PMC10610394; doi:10.3390/pharmaceutics15102468)
Supplement: Supplementary file 1 [file pharmaceutics-15-02468-s001.zip › pharmaceutics-2647663-supplementary.pdf]

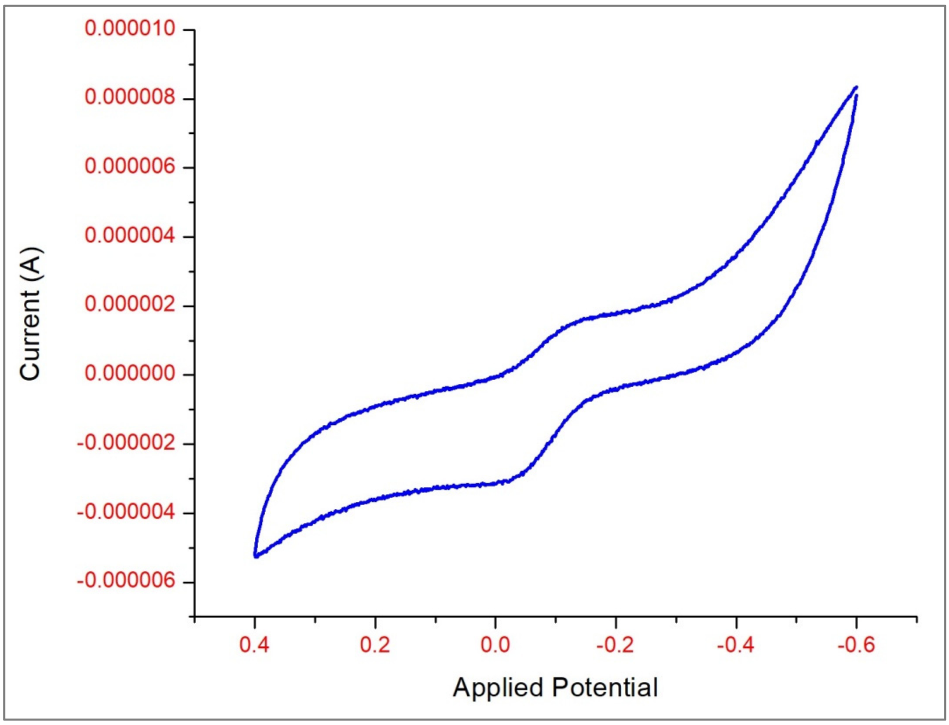


Figure S1. CV data of AgNPS synthesized using *H. arnottiana*.

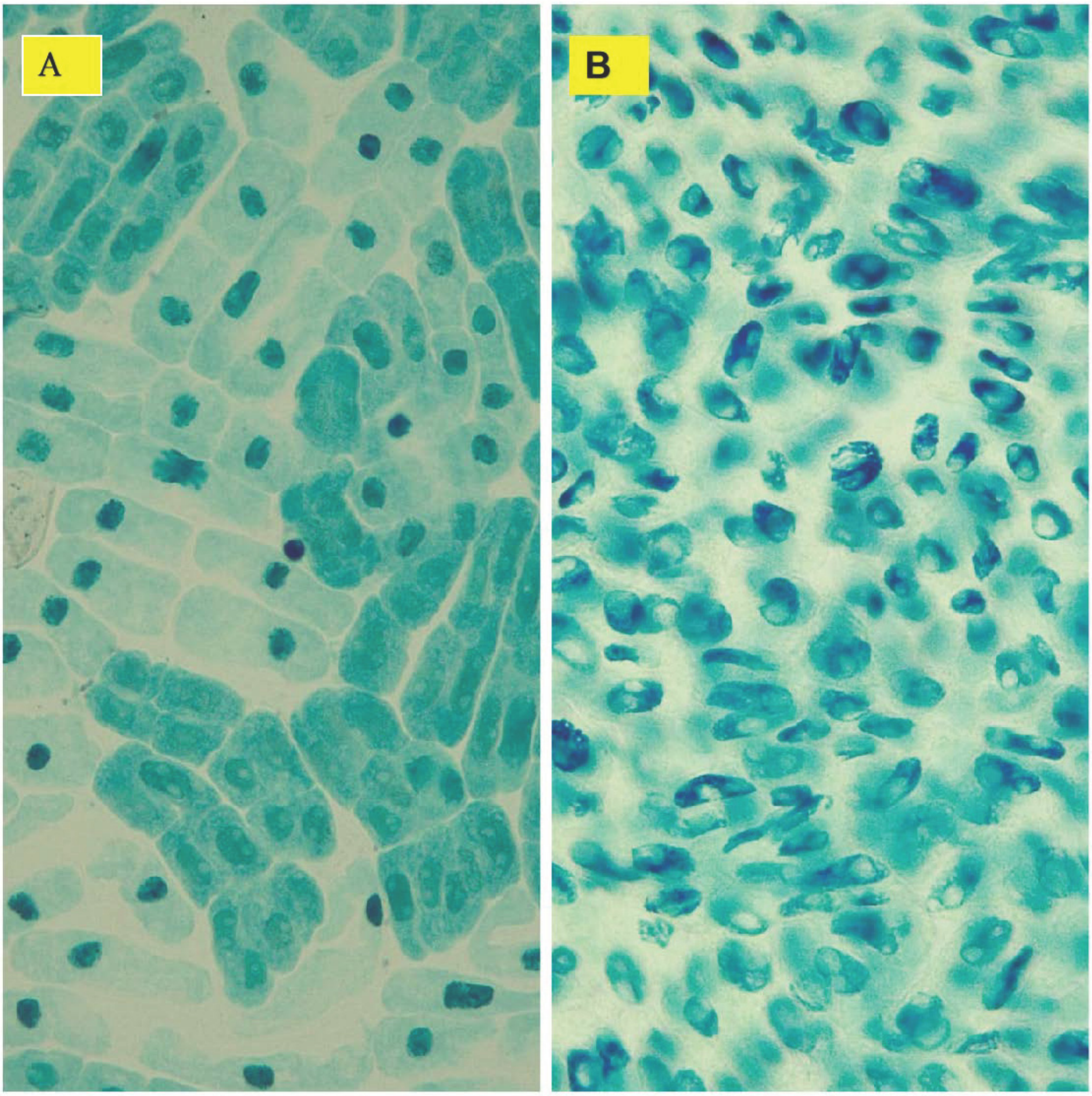


Figure S2: The Effect of Leaf Extract and Silver Nanoparticles on Mitotic Activity in Onion Root Tip Cells in the Context of Chromosomal Aberrations (A,B).

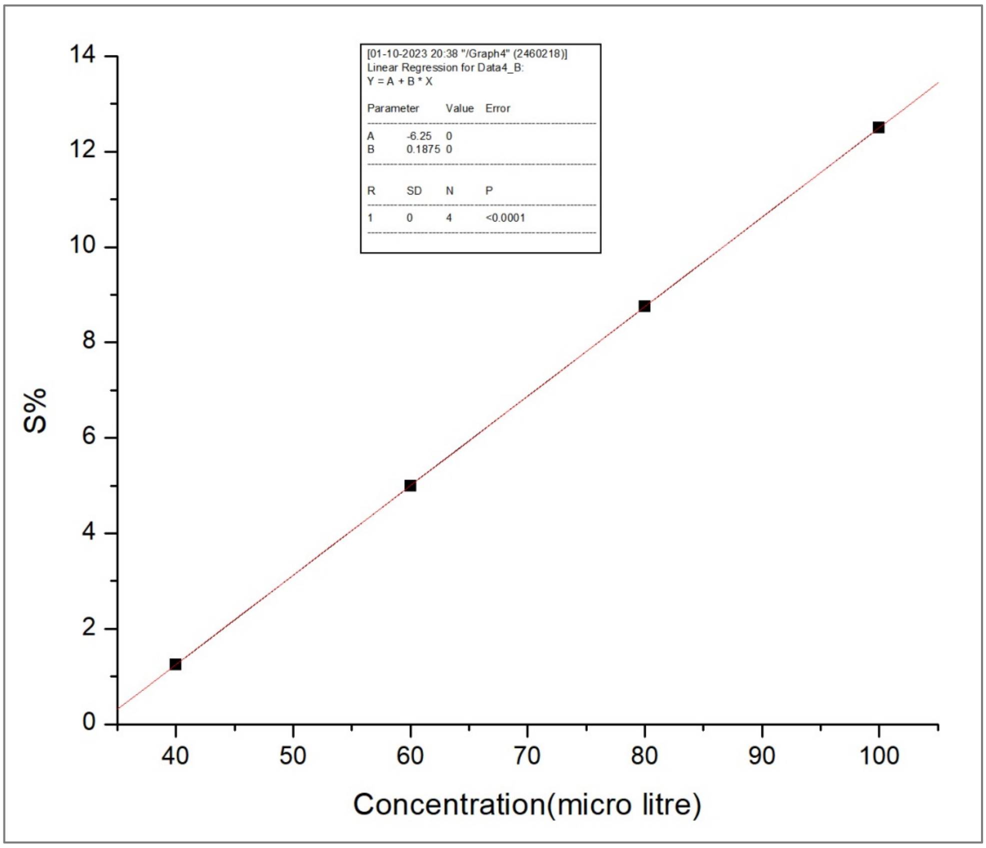


Figure S3. DPPH activity of leaf extract of *H. arnottiana*.
